# Amphiregulin and Fibrosis: Existing Evidence and Future Directions

**DOI:** 10.3390/ijms26167678

**Published:** 2025-08-08

**Authors:** Margherita Sisto, Sabrina Lisi

**Affiliations:** Department of Translational Biomedicine and Neuroscience (DiBraiN), Section of Human Anatomy and Histology, University of Bari “Aldo Moro”, I-70124 Bari, Italy; sabrina.lisi@uniba.it

**Keywords:** EGF, amphiregulin, autoimmune, fibrosis, EMT, TGF-β1

## Abstract

The fibrotic progression of diseases is characterized by the excessive deposition of extracellular matrix (ECM) proteins, leading to an alteration in tissue structure, often based on the activation of epithelial-to-mesenchymal transition (EMT). This can lead to decreased or completely impaired organ function, compromising quality of life and affecting vital organs. Fibrotic phenomena have recently been observed in autoimmune diseases and are correlated with the activation of transduction cascades that trigger chronic inflammation. Currently, effective therapeutic options remain limited due to the numerous molecular mechanisms that are activated and intersect with each other. Amphiregulin (AREG), a ligand for the epidermal growth factor receptor (EGFR), is involved in physiological cellular processes, but emerging data suggest that it plays a key role as a protein located at the crossroads of various activation mechanisms. The critical role of AREG as a molecular bridge between inflammatory and fibrotic mechanisms has aroused our interest in deepening our understanding of AREG involvement in the fibrotic processes identified, to date, in inflammatory autoimmune diseases. The aim of this review is to evaluate emerging targeted interventions to modulate AREG-mediated molecular pathways in fibrotic processes observed in autoimmune diseases, starting with the structure of AREG and the molecular mechanisms in which the protein is involved.

## 1. Introduction

Fibrotic autoimmune diseases are a group of severe chronic pathologies in which the body mistakenly attacks its own organs and in which fibrosis develops as the end result of a persistent inflammatory process [1,2,3]. These conditions are characterized by the involvement of the intense activation and proliferation of fibroblasts, which differentiate into myofibroblasts producing an excessive accumulation of extracellular matrix (ECM) proteins such as collagen and fibronectin, which lead to progressive tissue scarring and organ dysfunction [4,5]. In recent years, many studies been conducted, since these disorders were recognized to have a high impact on global morbidity and mortality rates, and there is an urgent need to identify the molecular mechanisms of the fibrogenesis and discover novel therapeutic targets.

Starting from these premises, recent investigations have evidenced the critical role of several growth factors and cytokines in orchestrating the complex cellular and molecular network that drives fibrotic mechanisms. Among these factors, amphiregulin (AREG), a member of the epidermal growth factor (EGF) family, is currently considered a key mediator capable of creating an intersection between mechanisms of tissue damage, chronic inflammation, and tissue regeneration, contributing to both physiological wound healing and pathological fibrosis [6,7,8].

AREG was discovered in human breast carcinoma cells in conditioned media and was originally described as a bifunctional factor of cell growth. It is able to inhibit the growth of tumoral cells and to stimulate the proliferation of fibroblasts [9].

Currently, several studies have highlighted the functional role of AREG in several aspects of carcinogenesis, including tissue invasion and metastasis [10,11]. In addition, AREG, through its binding and activation of the EGF receptor (EGFR), triggers complex signalling events, particularly in inflammatory and pathogenic conditions. Most recent findings have increasingly recognized the role of AREG in mechanisms of chronic autoimmune diseases characterized by a fibrotic evolution [12,13].

In this review, we will discuss the current understanding of the structure, as well as the regulatory and functional features, of AREG, and its strategic role in the fibrotic process observed in several autoimmune diseases.

## 2. *AREG* Gene and Protein

Structural and molecular evolutionary analyses have identified several structural characteristics that distinguish the *AREG* gene [9,14,15]. In humans, there are two copies of the gene, known as AREG and AREGB, that include about 10 kb of genomic DNA located on the q13–q21 region of chromosome 4 within the *EGF* family gene cluster. These two *AREG* genes were found to be approximately 160 kb apart and are flanked by the betacellulin (*BTC*) gene at the 3′ region and by the epiregulin (EREG) and epigen (*EPGN*) genes at the 5′ region, respectively [16]. AREG is transcribed as a 1.4 kb pre-protein mRNA transcript composed of six exons that are translated to a membrane-anchored precursor glycoprotein of 252 amino acids named pro-AREG [17].

**Figure 1:**
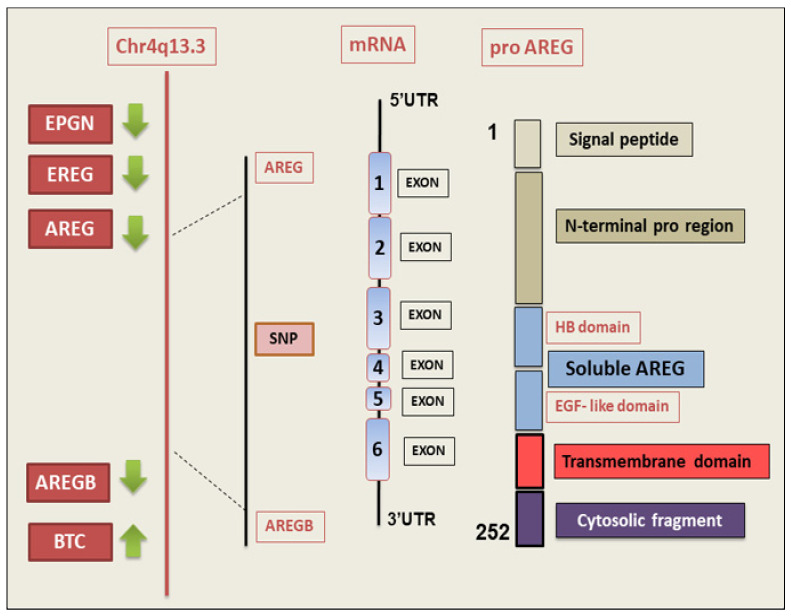
Structural characteristics of the human *AREG* gene and protein domains. The gene is drawn in 5′-to-3′ to scale. The chromosomal localization of *AREG* and *AREGB* genes is shown. The *AREG* gene is transcribed as a 1.4 kb mRNA constituted of six exons that code for an immature transmembrane glycoprotein, named pro-AREG, of 252 amino acids. BTC (betacellulin); EPGN (epigen); EREG (epiregulin); HB domain (heparin-binding domain); NLS (nuclear localization signal); SNPs (single-nucleotide polymorphisms); UTR (untranslated region).

Pro-AREG includes several domains: a hydrophobic signal peptide, a hydrophilic extracellular N-terminus with a glycosylation portion, a heparin-binding (HB) domain containing another glycosylation site and a nuclear localization signal, and the EGF-like domain with six spatially conserved cysteines responsible for disulfide bridges, followed by a juxta-membrane stalk containing the cleavage site for “AREG ectodomain shedding” [18]. The hydrophobic domain transverses the cell membrane and an intracellular cytoplasmic tail with another nuclear localization signal, a novel mono-Leu basolateral sorting motif [19], and a ubiquitination site at Lys240 implicated in AREG endocytosis [20]. Successively, at the plasma membrane, pro-AREG is subjected to sequential proteolytic activity within its ectodomain and is then cleared as the mature soluble AREG protein [10]. Mature soluble AREG, which includes the EGF module, is produced through proteolytic cleavage at the site (Lys187), a process known as “ectodomain shedding” of pro-AREG [18]. The cleavage of pro-AREG occurs at two N-terminal sites, which produce two major soluble forms of AREG (~19 and ~21 kDa). In addition, the ectodomain shedding of pro-AREG can give rise to a larger soluble protein of 43 kDa in proportion to the entire extracellular domain, which can be released into the extracellular milieu. Shedding of the extracellular pro-AREG domain can essentially be initiated by tumour necrosis factor-α converting enzyme (TACE), a member of the disintegrin and metalloproteinase (ADAM) family also referred to as ADAM17 [21]. Once this pro-AREG cleavage occurs, the mature protein is released, which induces the activation of its receptor EGFR on neighbouring cell membranes in an autocrine or paracrine manner [12,22]. The interaction between AREG and EGFR triggers intracellular signalling cascades, such as PI3K/Akt and the MAPK pathways, which promote cell proliferative and survival/antiapoptotic signals [18,23]. Sequential proteolysis at other alternative cleavage sites generates several active soluble forms of AREG containing the HB and/or the EGF domains [24]. Transmembrane Pro-AREG can also activate EGFR in a juxtacrine manner [25] (Figure 1).

## 3. AREG: Mechanism of Action

**Figure 2:**
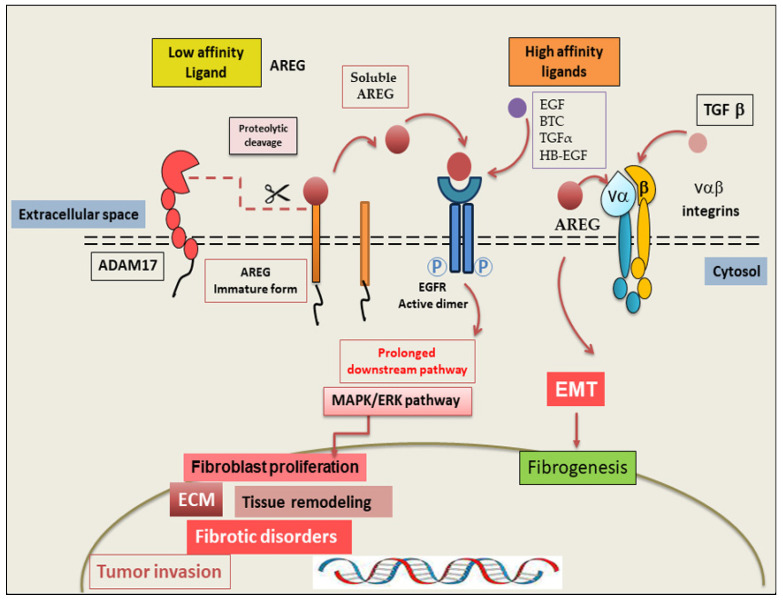
Schematic representation of the most common AREG signalling pathways. AREG is produced as an immature protein (pro-AREG), which is cleaved by the matrix metalloproteinase, ADAM-17. The soluble mature AREG is released into the microenvironment, in which it activates EGFR via autocrine and paracrine signalling. AREG-mediated EGFR signalling stimulates the MAPK/ERK pathway, promoting fibroblast differentiation, ECM accumulation, and fibrotic disorders. AREG can interact through EGFR-independent mechanisms, with αvβ integrins activating TGF-β, which triggers EMT, leading to the development of fibrotic disease. ADAM-17 (A disintegrin and metalloprotease-17); BTC (betacellulin); ECM (extracellular matrix); EGFR (epidermal growth factor receptor); EGF (epidermal growth factor); EMT (epithelial–mesenchymal transition); ERK (extracellular signal-regulated kinase); HB-EGF (heparin-binding EGF-like growth factor); MAPK (mitogen-activated protein kinase); TGF-β (transforming growth factor-β).

AREG is a low-affinity EGFR ligand that primarily acts by binding to a receptor in a competitive manner, inducing downstream signalling pathways involved in vital biological functions such as cell growth regulation, proliferation, and tissue differentiation [26]. Structurally, the receptor comprises an extracellular ligand-binding domain and an intracellular tyrosine kinase domain [22]. In its inactive form, the receptor exists primarily as a monomer [22]. Indeed, EGFR undergoes an important conformational change that allows it to dimerize. The dimerization process of the extracellular domains leads to the auto-transphosphorylation of the Tyr992 residue, thereby activating a complex network of pathways [27,28]. Interestingly, AREG acts as a partial agonist, triggering only about half as much total dimerization as the other three ligands—EGF, TGFα, and BTC—suggesting significant differences in biological response. However, sustained signalling activated by AREG and EGFR leads to the prolonged activation of various downstream pathways, particularly extracellular signal-regulated kinase (ERK)/MAPK cascades that drive persistent cellular activation and increased proliferation [29,30]. Indeed, in fibrotic diseases, this prolonged EGFR activation determines the continued stimulation and proliferation of fibroblasts and epithelial cells, triggering abnormal ECM protein deposition and tissue remodelling [13,31]. In addition, AREG works as a key amplifier of EGFR signalling, being able to integrate signals from other low-affinity EGFR ligands at the receptor state, generating multiple signalling modalities that induce pathological processes such as fibrotic disorders and tumour invasion [22,32]. Recent findings have provided evidence that AREG can exercise its pro-fibrotic role through EGFR-independent mechanisms, particularly via interactions with αvβ integrins activating TGF-β, creating a critical loop between the EGFR and TGF-β pathways [33,34]. Since AREG interacts with αvβ integrins, it is able to promote the activation of TGF-β, a master regulator of fibrosis. Interestingly, AREG stimulates the activation of the epithelial–mesenchymal transition (EMT) programme, downregulating epithelial cell marker expression and increasing the expression of mesenchymal markers; in this way, AREG drives the development of fibrotic disease and cancer invasion [35]. Emerging findings have evidenced that the formation of the soluble form of AREG depends on the proteolytic event of the proteinase ADAM17. Many physiological and pharmacological agonists, including TLR, TKR, and G-protein, when using binding rather than receptors, are able to modulate the activity of ADAM17 and promote EGFR transactivation [36]. The release of mature AREG can be achieved using several cytokines, UV radiation, and chemotherapeutic drugs, among other mechanisms [32]. As mentioned before, AREG possesses a double signalling ability, through both EGFR-dependent and EGFR-independent pathways, and this intriguing role of AREG makes it a critical mediator of both intrinsic- and acquired-resistance mechanisms in EGFR-driven diseases [13]. A prospective summary of the AREG-mediated fibrotic mechanisms is shown in Figure 2.

## 4. AREG as a Driver of Fibrotic Mechanisms

The relationship between fibrosis and inflammation has been increasingly discussed in recent decades, and recent investigations revealed a critical role of AREG in orchestrating the complex cellular and molecular interactions that drive fibrotic processes [37]. Findings have demonstrated that elevated AREG expression level was correlated with disease severity across multiple organs, including the lungs, kidneys, liver, and heart. High levels of AREG are also present in alveolar stem cells of mouse fibrotic lungs [6] and in the cardiac tissue of mice showing experimental EGFR activation-dependent myocardial infarction [38]. Confirming these data, the block of AREG signalling determines a strong reduction in fibrotic outcomes [39]. Recent studies have revealed an involvement of AREG in the development of fibrosis following total body irradiation (TBI) [40]. Shao et al. suggested that AREG is a key mediator of fibrogenesis in intestinal epithelial growth after TBI treatment. Accordingly, the expression of the *Areg* gene was elevated in the intestines of mice after irradiation, and the use of small interfering RNA (RNAi) technology targeting mouse Areg mRNA was shown to lead to a reduction in radiation-induced organ damage, particularly in fibrotic organs such as lungs and kidneys [40,41]. Other experimental evidence has hinted that AREG promotes fibroblast differentiation in liver fibrosis in the atherosclerosis process [42]. Also, in this case, *Areg* gene-silencing using RNAi was sufficient to inhibit fibroblast proliferation and to reduce collagen accumulation in the lungs of TGF-β1 transgenic mice [43]. These investigations showed an important regulatory activity of AREG in the pathogenesis of TGF-β1-induced pulmonary fibrosis. Indeed, TGF-β1 is a key upstream regulator that induces AREG expression in epithelial cells, causing an interesting loop that amplifies the fibrotic mechanism. This persistent AREG signalling transforms the physiological event of repair into pathological fibrosis [42]. Wang et al., in a previously published paper, identified elevated levels of AREG in intestinal biopsy tissues and Th17 cells in the peripheral blood of Crohn’s disease (CD) patients with severe intestinal fibrosis [44]. Recently, the research group induced chronic colitis in both wild-type and Areg-knockout mice, demonstrating the role of AREG as a key regulator bridging tissue injury to the development of intestinal fibrosis [42]. Therefore, exogenous AREG administration exacerbates intestinal fibrosis in mice, as evidenced by increased collagen deposition and upregulated collagen gene expression. Conversely, using *Areg* gene knockout mice, the animal model shows a marked improvement in fibrosis, confirming the key role of AREG in driving intestinal fibrosis [42]. A similar trend was found in patients with CD, in whom a high concentration of AREG was found in fibrotic regions, and particularly in fibroblasts isolated from stenotic sites [42]. This study evidenced the pro-fibrotic role of AREG in the pathogenesis of intestinal fibrosis, mediated across the PI3K/AKT pathway, as demonstrated in both in vitro and in vivo models. Specific inhibitors of the PI3K/AKT pathway demonstrated a significant attenuating effect on AREG-induced intestinal fibrosis, supported by reduced collagen deposition. Other studies revealed that AREG increases the proliferation of hepatic stellate cells (HSCs) through mitogenic signalling pathways such as PI3K and p38 [45,46], whereas a protective role of AREG as a signal transducer and activator of transcription 1-dependent apoptosis of HSCs was also reported [47]. Interestingly, investigations suggest that AREG activates fibroblasts, also triggering downstream pathways MAPK/ERK and Smad, promoting fibronectin, collagen, and ECM deposition [48,49]; in this way, AREG stimulates fibroblast proliferation, migration, and differentiation into myofibroblasts, further exacerbating tissue remodelling and fibrosis [48]. In accordance with the datum that AREG is an important driver for myofibroblast differentiation under severe inflammatory conditions, it was also shown that AREG-deficient mice are resistant to the development of tissue fibrosis. In chronic kidney disease, renal interstitial fibrosis is prevalent and often leads to organ failure and death of patients. In this context, the differentiation of fibroblasts into myofibroblasts in the renal interstitial space is a major cause of fibrosis. Son and collaborators demonstrated that AREG is upregulated in mouse and human proximal tubule cells. The authors reported that, using the novel approach represented by the AREG-targeting Self-Assembled-Micelle inhibitory RNA (SAMiRNA-AREG), the stable silencing of the *Areg* gene reduces fibrotic processes and alleviates the side effects of conventional siRNA treatment of fibrosis in kidney disease models [50]. Furthermore, the same authors evaluated the effects of SAMiRNA-AREG in human and mouse proximal tubular cells and mouse fibroblasts following stimulation with TGF-β1, clearly demonstrating the inhibitory and anti-fibrotic effect of this treatment. Finally, SAMiRNA-AREG represents a novel siRNA therapeutic approach for renal fibrosis by suppressing EGFR signals [50]. Several investigations have corroborated the fact that the AREG signalling pathway also involves upstream inductors such as ADAM17, which promote the EGFR-AREG axis, leading to an increase in markers characteristic of the EMT processes [49,51].

**Figure 3:**
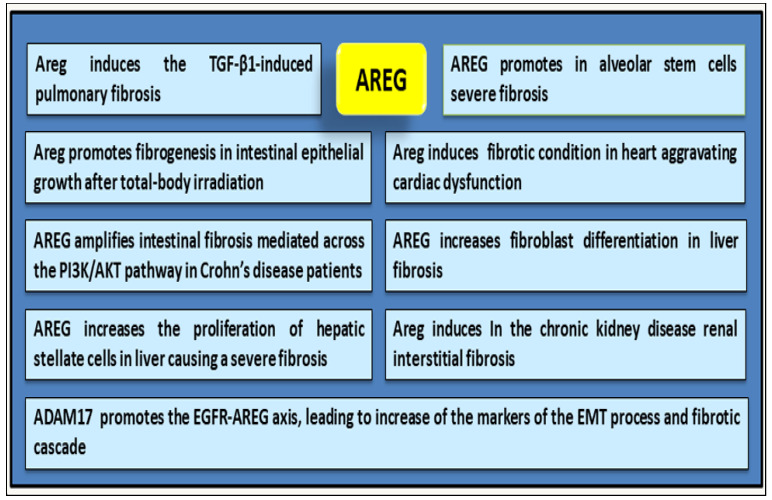
Scheme of AREG activity in fibrogenesis.

Since AREG is considered a critical component in fibrotic cascades, it makes it a promising and potential target for fibrotic disorders and fibrosis-associated inflammatory processes, as well as serving as a biomarker for evaluating the disease progression. A schematic summary of the role played by AREG in the activation of fibrosis is shown in Figure 3.

## 5. Analysis of the Correlation Between AREG Expression and Fibrosis in Autoimmune Diseases

In recent years, numerous pieces of evidence have been collected regarding the involvement of AREG in the activation of a fibrotic process in autoimmune diseases. This does not seem surprising, since autoimmune diseases are characterized by chronic inflammation and a close correlation has been demonstrated between a state of high and long-lasting inflammation, autoimmunity, and fibrosis [52,53,54]. One of the main activation cascade mechanisms in which AREG is involved is mediated through the activation of TGF-β1, a major player in the triggering of fibrotic processes [33]. Since TGF-β1 is widely considered a central mediator in the phases of tissue architecture modification and remodelling that, under certain circumstances, can lead to the development of fibrotic processes, the canonical TGF-β1 activation cascade pathway involves the activation of the receptor followed by the phosphorylation and activation of Smad proteins that translocate to the nucleus, determining the transcription of numerous TGF-β1-specific target genes [55]. TGF-β1 is also known to activate non-canonical signalling pathways, among which there are some that are widely studied and involved in numerous pathogenetic processes, such as PI3K/Akt and MAPK signalling [56]. As often happens, the canonical and non-canonical activation pathways communicate with each other through various common factors, with different effects on cells and tissues [57].

### 5.1. The Role of AREG in IPF-Related Fibrosis

**Figure 4:**
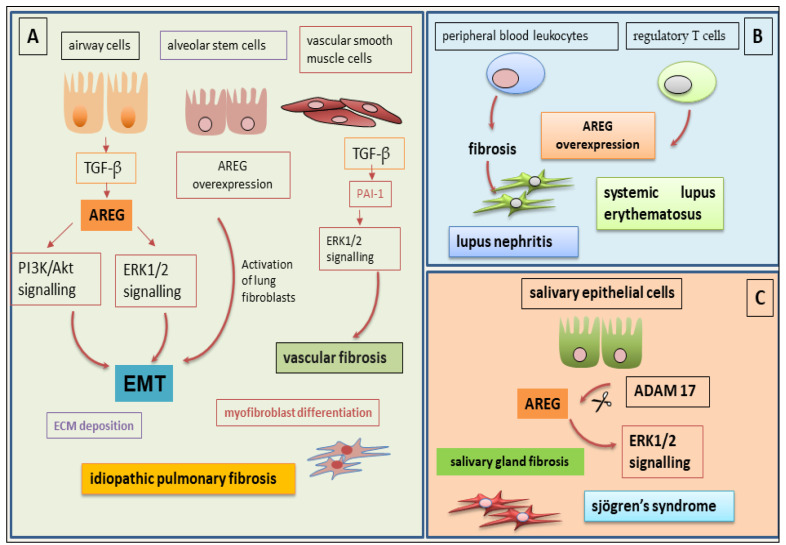
Signal transduction pathways of AREG in autoimmune disease-related fibrosis. AREG signalling in idiopathic pulmonary fibrosis (**A**), systemic lupus erythematosus (**B**), and in Sjögren’s syndrome (**C**). ADAM-17 (A disintegrin and metalloprotease-17); ECM (extracellular matrix); EMT (epithelial–mesenchymal transition); ERK (extracellular signal-regulated kinase); PAI-1 (plasminogen activator inhibitor-1); PI3K (phosphoinositide 3-kinase); TGF-β (transforming growth factor-β).

Studies of the autoimmune disease idiopathic pulmonary fibrosis (IPF) have revealed that the activation of signal transduction pathways mediated by the activation of PI3K/Akt and ERK1/2 MAPK is the basis of the phenomena of airway remodelling induced by TGF-β. This activation occurs through the activation of the EMT programme. This process involves the transformation of myofibroblasts and an abundant accumulation of collagen in the lung [58,59,60], a symptom of the ongoing fibrotic process. Recent studies have clarified the role played by the EGFR-mediated signalling pathway in these mechanisms. The signal activated through EGFR activation determines the TGF-β1-induced transcription of plasminogen activator inhibitor-1 in vascular smooth muscle cells [61] and TGF-β1-induced expression of COX-2 in human bronchial epithelial cells [62]. In this context, what role is attributed to AREG? AREG activation is induced by TGF-β1 through the activation of the EGFR-dependent canonical and non-canonical pathways. The fundamental role of AREG in mediating EGFR activation is essential for TGF-β1 to perform its effector function during the fibrogenesis process; in fact, siRNA-mediated *AREG* gene-silencing experiments and the chemical inhibition of EGFR signalling lead to a significant reduction in fibroblast proliferation, a reduction in EMT-mediated myofibroblast transformation, and a decrease in collagen accumulation in the lung. In IPF, the role of AREG reflects what has just been reported, namely that AREG stimulates the proliferation of lung fibroblasts [63] through its TGF-β1-mediated activation, resulting in myofibroblast transformation and the accumulation of ECM proteins. AREG, as a member of the EGF family, is also able to perform its function through binding to other EGFR ligands, such as TGF-α, EGF, HB-EGF, betacellulin, and epiregulin. Under physiological conditions, the triggering of these activation pathways regulates the proliferation and differentiation of lung epithelial and mesenchymal cells; stimulation by AREG specifically affects the development of different cell types that will compose the complex lung tissue [64]. While the role of AREG in the physiological development of lung tissue is sufficiently clear, the specific role of AREG in the pathogenesis of IPF is still debated, and the results obtained are controversial. Using the drug gefitinib, a specific inhibitor of EGFR, significantly reduces pulmonary fibrosis induced by bleomycin in vitro [65]; however, other authors have revealed the opposite effect, demonstrating that the administration of recombinant AREG reduces the inflammatory markers and pulmonary fibrosis induced by bleomycin, suggesting a potential protective role of AREG against tissue fibrosis [66]. These contradictory results could find an explanation in the heterogeneity of target organs, cell types, and injuries that could trigger the fibrotic process, considering the harmful consequences of treatment with bleomycin. In an attempt to clarify which mechanisms can explain the complex interaction between AREG, TGF-β1, and pulmonary fibrosis, a mouse model was developed in which the biologically active human TGF-β1 is specifically overexpressed in the murine lung [58]. The results obtained have been very interesting. In this genetically engineered mouse model, a pathology resembling human pulmonary fibrosis develops, characterized by inflammation, the development of fibrotic material in the airways and lung parenchyma, hyperplasia of myocytes and myofibroblasts, and remodelling of the alveolar architecture [55,58,67,68]. This mouse model with stringent characteristics for the lung has allowed us to demonstrate the crucial role of AREG and EGFR signalling in TGF-β1-induced pulmonary fibrosis; in this mouse model, both *AREG* gene-silencing and the use of specific EGFR inhibitors led to a significant reduction in TGF-β1-induced pulmonary fibrosis. However, we are still far from being able to use these EGFR inhibitors in clinical practice because they have toxic side effects whose pathogenetic mechanisms are still to be understood [69]. Given the adverse events that hinder the use of EGFR inhibitors in clinical practice in cases of pulmonary fibrosis, AREG inhibition could represent a valid alternative to control the evolution of the fibrotic process in cases of chronic inflammatory diseases with a marked autoimmune component, such as IPF. Recently reported and very interesting data predicts there is a correlation between AREG overexpression in AT2 intermediate cells of the lung parenchyma, their distribution pattern, and the development of fibrotic tissue in an experimental model of pulmonary fibrosis [6]. Comparing the expression levels of AREG in fibrotic mouse lungs with those found in human lungs with IPF, a clear homogeneity of expression was demonstrated. Through performing experiments involving the silencing and subsequent reactivation of AREG, it was concluded that AREG in AT2 intermediate cells is responsible for the activation of EGFR-mediated fibrosis through the activation of lung fibroblasts. Furthermore, elevated levels of AREG found in the peripheral circulation of IPF patients have been closely correlated with loss of lung function. From a therapeutic point of view, it has been seen that using a neutralizing antibody against AREG resulted in a clear improvement in lung functionality, with a reduction or even blocking of the progression of the fibrotic process. Of course, this was demonstrated in experimental murine models, but it suggests the possibility of a therapeutic process in IPF that is based on the use of anti-AREG antibodies that would act on AT2-type intermediate stem cells [6]. Recent studies suggest that the expression of AREG is correlated with disease severity in IPF patients. Some progress has been made regarding the correlation between AREG expression levels and the decline in lung function in patients with IPF through analyzing the correlation with he mortality levels in IPF patients, as measured by the GAP (Global Alignment and Proportion) score, a prognostic index of the disease. This data is also associated with the demonstration of a negative correlation between the level of EGFR mRNA and the severity of lung function parameters in IPF [70]. These data, taken together, seem to point towards the potential to use AREG levels as an indicator of the severity of disease in these IPF patients, suggesting the possibility of using them to monitor the progression of the disease (Figure 4).

The possibility of identifying the specific molecules to be used as AREG inhibitors in humans does not seem to be a far-fetched idea since, at least in the murine model, it was observed that mice genetically deficient in the *AREG* gene are nevertheless fertile and vital [71,72]. Furthermore, studies based on the gene-silencing of AREG by siRNA have obtained promising results both in mice and in primates [73,74,75], which did not present adverse side effects following the inhibition of AREG expression. Overall, these data motivate researchers to clinically develop an anti-AREG therapy that interferes with the AREG–EGFR axis and, in this way, circumvents the very negative effects of direct EGFR inhibition, hopefully leading to a reduction in the fibrotic picture in IPF patients. Obviously, further experimental data and validation are necessary.

### 5.2. AREG at the Basis of Fibrotic Phenomena in SLE

Systemic lupus erythematosus (SLE) is an autoimmune disease characterized by high morbidity and mortality, and these characteristics are inversely correlated with the age of the patients [76]. This disease is associated with inflammatory problems affecting various organs, and renal involvement, known as lupus nephritis (LN), significantly worsens the clinical picture and prognosis [77]. Currently, the mechanisms underlying LN remain unknown, despite the efforts of researchers [78]. Due to uncertainty related to the mechanisms involved in LN, the therapeutic prospects currently remain very poor; they cause significant adverse events and are often systemic and not very specific [77,78]. The need to identify new therapeutic targets has led to consideration of AREG, which has emerged in various experimental investigations as a crucial factor in the pathways that lead to chronic inflammation and the development of fibrotic tissue. AREG appears to be overexpressed in peripheral blood leukocytes of SLE patients [79]. Recently, a protective effect of AREG was demonstrated in an experimental model of LN. In this context of experimental LN induced by pristane oil treatment, a protective role of AREG was demonstrated, which seems to occur through the reduced activation of CD4+ T lymphocytes [78]. However, even in this autoimmune disease, AREG seems to have potent but sometimes opposite effects depending on the type of inflammation. It is tempting to speculate that during acute inflammatory processes, AREG acts in a pro-inflammatory way via the activation of monocytes or macrophages; on the other hand, in a situation of chronic inflammation, as is the case in LN, AREG might exert an anti-inflammatory activity, downregulating adaptive T cell immunity. AREG, therefore, exhibits anti-inflammatory and tissue-repairing protective functions but also properties that exacerbate the inflammatory picture, leading to a fibrotic evolution of the tissue. These different functions of AREG seem to be linked to the cell type involved in AREG secretion. When AREG is produced by macrophages—for example, in cases of uveitis [80]—AREG shows a protective and anti-inflammatory role. This macrophage production has been extensively studied by Minutti et al., demonstrating, once again, AREG-dependent TGF-β1 activation [80]. A tissue-repairing, and not immunosuppressive, effect was also demonstrated when AREG is produced by regulatory T cells (Treg), as shown in experimental models of muscle and lung injury [81,82]. This concept has recently been challenged by a study showing that Treg-derived AREG, which had been assumed to play a positive role in tissue repair, instead appears to induce and enhance collagen deposition and liver fibrosis in cases of non-alcoholic steatosis [7]. To further complicate the scenario, it has been shown that when AREG is secreted by different innate cells, such as macrophages, mast cells, and basophils, it significantly increases immunosuppressive functions; therefore, the different mechanisms activated depending on the cells involved remain unclear [83,84]. On the other hand, the pro-inflammatory role of AREG in various autoimmune diseases, such as psoriasis, rheumatoid arthritis, Sjögren’s syndrome, and allergic asthma, has been widely demonstrated. In all these diseases, the overexpression of AREG determines an increase in the production of pro-inflammatory cytokines, worsening tissue damage and predisposing the involved tissue to fibrotic evolution [85,86,87]. As in the case of SLE-associated LN, the influence of AREG on the fibrotic evolution of fibrosis has also been demonstrated in the kidney. In fact, the increased expression of AREG is significantly correlated with the severity of the fibrotic picture in both acute and chronic renal diseases [88]. This data is further supported by the observation that selectively inhibiting the *AREG* gene in proximal tubular cells leads to a decrease in pro-fibrotic parameters in murine models of unilateral ureteral obstruction and ischemia–reperfusion injury [7]. It remains, therefore, essential to analyze the specific roles played by the different cell types capable of producing AREG in LN, with the aim of obtaining useful information for the development of effective anti-AREG therapies (Figure 4).

### 5.3. The Debated Role of AREG in Salivary Gland Fibrosis in SjD

Sjögren’s disease (SjD) is a long-term, chronic inflammatory autoimmune disease that primarily affects the exocrine glands, particularly the lachrymal and salivary glands [89]. Common symptoms include dry mouth and dry eyes and other organ systems, such as the lungs, kidneys, and nervous system, are often severely affected. In such cases, the disease is indicated as secondary SjD [89]. It is now widely demonstrated that the EGF/EGFR system exerts significant effects on the regulation of cellular activities in the epithelial cells of SGs derived from patients with SjD [90,91] and is highly expressed in epithelial ductal cells, particularly in correspondence with the accumulation of lymphocytes characterized by marked tissue destruction. Once again, even in this autoimmune pathology, the EGF/EGFR system seems to play a positive or negative role depending on the molecular and tissue context analyzed; it follows that EGF is a key growth factor in the antiapoptotic and defence mechanisms activated in epithelial cells of SGs derived from patients with SjD [91]. In this context, the defence mechanism is based on the upregulation of the EGF/EGFR system expression, which, in turn, determines the activation of the classical intracellular anti-apoptogenic PI3K/Akt/IκB/NF-κB pathway [92,93]. In addition to these findings, the role of EGF/EGFR in SjD has been further explored from an experimental point of view, demonstrating that EGFR is a potent activator of the ERK1/ERK2 pathway, also known as the MAPK3/MAPK1 pathway, involved in the pro-inflammatory mechanisms that characterize the disease [94]. AREG’s role in SjD occurs in the transduction pathway that leads to the activation of the downstream effectors ERK1/2. The activation of AREG is subordinated to the activation of ADAM17 and to the transactivation of EGFR. These observations have been supported by inhibitory experiments of AREG blockade [95]. Most of the data supporting the relevance of the EGF/EGFR system in SjD comes from experiments showing a strong overexpression of AREG in SG biopsies of patients with SjD [95] and a concomitant high expression of active ADAM17 [96]. The data obtained on the expression of AREG in epithelial cells of SG of SjD were supported by the results of Kawasaki et al. [97], who demonstrated an upregulated gene expression of AREG in the conjunctival epithelium of patients with SS. Based on these assumptions, recent research has evaluated the correlation between the ADAM17-dependent activation of AREG and the fibrotic evolution of SGs that is observed with increases in the degree of inflammation. It was observed that, with the progression of SjD, chronic inflammation leads to irreversible SGs fibrosis, mediated mainly by the M2-type macrophage [98]. The activator of these pro-fibrotic mechanisms appears to be TGF-β1, through both the canonical SMAD-mediated pathway and non-canonical activation pathways. It has been demonstrated that TGF-β1 facilitates the polarization of M2 macrophages by activating the trimeric SMAD2/3/4 complex, and this also determines the promotion of fibrotic mechanisms through the conversion of fibroblasts into myofibroblasts [99]. As reported in the previous paragraphs, TGF-β1 is a major factor in fibrosis in many chronic inflammatory diseases [100]. Recently, the EMT mechanism was identified as the basis of the fibrotic evolution of the salivary glands associated with a higher degree of inflammation, a significant pathological response of SG epithelial cells to chronic inflammation in SjD. Indeed, a strong positive expression of EMT-related proteins (vimentin, α-SMA, collagen type I and Snail) has been demonstrated in the SGs of SjD patients [101], and TGF-β1 was shown to initiate the EMT programme through the classical TGF-β1/SMAD/Snail-signalling pathway [101]. Currently, however, the direct correlation between AREG activation and fibrotic progression of SGs tissue in SjD requires further research. All these findings raise the possibility that specific EGF/EGFR/ADAM17/AREG inhibitors may be of therapeutic value for treating the chronic inflammation characterizing SjD disease, but, due to the dual role played by these factors depending on the cellular and tissue context considered, this possibility remains fascinating but far from realization (Figure 4).

## 6. Potential Application of Innovative Anti-AREG Therapies

RNA interference (RNAi) technology has been developed to block the translation of mRNA into proteins using the complementarity between mRNA and siRNA sequences [40,41]. In this way, specific siRNAs can be constructed for protein targets linked to or involved in the development of various diseases; this represents a very recent field of investigation [40,41]. Although siRNA technology represents a valid approach to modulate gene expression both in vitro and in vivo, a major limitation, which slows down the possibility of its therapeutic use, is represented by the nonspecific immunostimulatory function exhibited by siRNAs [40,41,43]. To improve this method of gene transcription inhibition, a new siRNA molecule was recently developed by conjugating it with a hydrophobic hydrocarbon and a hydrophilic polymer at the 5′ and 3′ ends, respectively. These siRNA molecules have the tendency to form micelles spontaneously, without the addition of other chemicals, and have been defined as self-assembled micellar inhibitory RNAs (SAMiRNAs). The advantage of SAMiRNAs is that they do not trigger innate immune responses [102]. The use of SAmiRNAs has also gained ground in the therapeutic field of fibrotic diseases, because SAMiRNAs penetrate and are translocated by mesenchymal cells, such as fibroblasts and myofibroblasts, and by inflammatory cells, whose intercellular communication triggers the main mechanisms of fibrotic transformation and evolution. This phenomenon has been extensively experimentally evaluated in IPF, where SAMiRNAs were found to be able to penetrate key cells responsible for lung fibrosis, as well as inflammatory and structural cells involved in the fibrotic evolution of lung tissue [103]. SAMiRNAs also have the significant advantage of not undergoing structural changes and of having high stability, even over many months. Given the fundamental role played by TGF-β in regulating cellular processes that, if altered, trigger fibrotic transformation through various molecular activation pathways, several therapeutic strategies aimed at blocking molecules involved in TGF-β-mediated fibrosis processes have been evaluated. Among these mediators, AREG has, in recent years, gained great appeal as a molecular target whose modulation could represent an alternative and effective method to block pulmonary fibrosis. Researchers designed SAMiRNAs targeting mouse Areg mRNA (SAMiRNA-mAREG) and, in this experimental model, after inoculation into the target organs, the SAMiRNAs achieved the targeted inhibition of AREG synthesis and a consequent decrease in all parameters associated with pulmonary and renal fibrosis [50]. Interestingly, greater efficacy in restoring lung function and decreasing fibrosis markers, such as collagen deposition or other ECM proteins, was demonstrated in the mouse model of IPF following the synergistic use of SAMiRNAs specific for AREG and connective tissue growth factor (CTGF) compared to the use of SAMiRNAs for AREG or CTGF alone. These results suggest a potential combined action of AREG and CTGF in driving the initiation and perpetuation of fibrotic phenomena in the lung [103]. This hypothesis seems to be supported by the observation that CTGF is a ligand for EGFR, whose activation is mediated by TGF-β [104]. Therefore, it seems plausible that both AREG and CTGF may be required to optimally activate the EGFR/TGF-β-mediated pulmonary fibrosis pathways [104]. Unfortunately, we are still far from testing these inhibitors in humans, and the interaction between AREG and CTGF also requires further experimental investigation before being fully demonstrated. The therapeutic effect of SAMiRNA-AREG, reported in mouse models and characterized by the stable silencing of the *AREG* gene, resulting in a reduction in the side effects of conventional siRNA treatment of pulmonary fibrosis, has also been evaluated in renal fibrosis [50]. In mouse models of renal fibrotic disease, the protective effect of SAMiRNA-AREG was confirmed. SAMiRNA-AREG significantly reduced AREG mRNA expression and the expression of all fibrosis markers. Simultaneously, the transcription of inflammatory markers, which are often elevated in fibrotic processes, was attenuated. This same result was obtained by analyzing a radiation-induced fibrotic process, in which treatment with SAMiRNA-mAREG significantly inhibited the expression of AREG and markers of TGF-beta-dependent fibrosis in the kidneys. These data are very useful in the attempt to develop strategies to alleviate radiation-induced fibrosis following radiotherapy treatments or after accidental exposure to radiation [50]. A traditional, but equally promising, therapeutic approach is based on the use of AREG-specific neutralizing antibodies. The rationale for this use is based on experimental evidence that AREG-EGFR signalling plays a role in promoting pulmonary fibrosis. To date, EGFR inhibitors have shown a promising attenuation of fibrosis in various animal models of IPF [6], but this is compromised by their high pulmonary toxicity. This has led researchers to search for new inhibition modalities, focusing their attention on the use of AREG-specific Abs, which are generally well tolerated in murine models; these experiments have been supported by the observation that Areg-null mice are fertile and have a comparable life expectancy to non-genetically modified mice [105]. The obvious limitations of the reported studies are the lack of human data, with all that this entails (an analysis of different demographic characteristics and the various clinical stages of different autoimmune and non-autoimmune diseases); however, the potential applicability of AREG inhibition is increasingly being strengthened and could lead to AREG being considered for use as an indicator of the severity of diseases characterized by fibrotic evolution.

## 7. Conclusions

It is now widely recognized that chronic inflammation can predispose to organ fibrosis; therefore, the release of pro-inflammatory cytokines and the activation of fibroblasts and epithelial cells with EMT phenotypes have been implicated in several autoimmune diseases. Recent research has uncovered a key role for AREG as a pivotal molecule around which various transduction cascades revolve, leading to the activation of specific genes. It is fascinating to delve into their various activation mechanisms and try to identify common molecules. However, could this have therapeutic value, which is the goal of experimental research in the clinical setting? Currently, the primary need appears to be to quantify AREG in order to develop a reference scale for various autoimmune diseases. Furthermore, it is essential to evaluate the efficacy and safety of the experimental therapies identified in order to transition AREG inhibition from a subject of preclinical research to a method with clinical application. Finally, the experimental data currently available on AREG in autoimmune diseases are limited to a small number of conditions. Future clinical validation will require studies conducted in patients who also suffer from other autoimmune diseases. Such studies will likely support future advances in the treatment of autoimmune diseases with fibrotic progression and the development of interesting and innovative therapeutic approaches.
